# Novel Zinc-Catalytic Systems for Ring-Opening Polymerization of ε-Caprolactone

**DOI:** 10.3390/molecules20022816

**Published:** 2015-02-09

**Authors:** Karolina Żółtowska, Marcin Sobczak, Ewa Olędzka

**Affiliations:** 1 Department of Inorganic and Analytical Chemistry, Faculty of Pharmacy, Medical University of Warsaw, ul. Banacha 1, 02-097 Warsaw, Poland; E-Mails: karolina_zoltowska@o2.pl (K.Z.); eoledzka@wp.pl (E.O.); 2 Chair of Chemistry, Department of Organic Chemistry, Faculty of Materials Science and Design, Kazimierz Pulaski University of Technology and Humanities in Radom, ul. Chrobrego 27, 26-600 Radom, Poland

**Keywords:** poly(ε-caprolactone), aliphatic polyesters, biomedical polymers, ring-opening polymerization, diethylzinc

## Abstract

Polycaprolactone (PCL) is a biodegradable synthetic polymer that is currently widely used in many pharmaceutical and medical applications. In this paper we describe the coordination ring-opening polymerization of ε-caprolactone in the presence of two newly synthesized catalytic systems: diethylzinc/gallic acid and diethylzinc/propyl gallate. The chemical structures of the obtained PCLs were characterized by ^1^H- or ^13^C-NMR, FTIR spectroscopy and MALDI TOF mass spectrometry. The average molecular weight of the resulting polyesters was analysed by gel permeation chromatography and a viscosity method. The effects of temperature, reaction time and type of catalytic system on the polymerization process were examined. Linear PCLs with defined average molecular weight were successfully obtained. Importantly, in some cases the presence of macrocyclic products was not observed during the polymerization process. This study provides an effective method for the synthesis of biodegradable polyesters for medical and pharmaceutical applications due to the fact that gallic acid/propyl gallate are commonly used in the pharmaceutical industry.

## 1. Introduction

Over the last decade, biodegradable or bioresorbable polymers have been widely employed in biomedical applications such as implants, sutures, tissue scaffolds or various drug delivery systems [1,2,3,4]. The most commonly used synthetic biodegradable polymers for medical and pharmaceutical applications are aliphatic polyesters (e.g., poly(ε-caprolactone), polyglycolide, polylactide or co- or terpolymers of ε-caprolactone, glycolide, l-lactide and *rac*-lactide) [5,6,7,8]. 

Polycaprolactone (PCL) is an important biomedical polyester due to its physicochemical properties as well as good biocompatibility and biodegradability [9,10]. PCL is a hydrophobic and semi-crystalline polymer. It was one of the earliest polymers synthesized, by the Carothers group in the early 1930s [9]. PCL can biodegrade in a time-span ranging from several months to several years, depending on its molecular weight, degree of crystallinity and the condition of biodegradation process [10]. Due to its multiple biomedical applications, the synthesis of PCL has received increased attention in the last years [9,10]. There are two methods for the preparation of biomedical PCLs: the polycondensation of 6-hydroxyhexanoic acid and the ring-opening polymerization (ROP) of ε-caprolactone (CL) [9,11]. The ROP process is more often used because of the higher molecular weight and lower polydispersity of the obtained polyesters [9]. The ROP of CL can be carried out in the presence of anionic or cationic initiators and coordinating or enzymatic catalysts [7,8,9,12]. A wide range of metals have so far been studied for the ROP of CL through the coordination-insertion mechanism (e.g., aluminium, calcium, iron, magnesium, tin, titanium, zinc, lanthanides and rare earth metals) [13].

Zinc-based catalysts have been reported to be very effective initiators of the ROP of CL, resulting in a degree of polymerization of over 100 and a polydispersity index (*PD*) between 1.05 and 1.1. In this case, the ROP was carried out through a coordination–insertion mechanism relying on the cleavage of the acyl-oxygen bond of CL [9]. The polymerization mechanism involves the coordination of the CL molecule to the Lewis acidic-metal centre through the exocyclic oxygen. The carbonyl group of the lactone is therefore rendered more susceptible to nucleophilic attack [13]. It is known that a high temperature and long reaction time foster the inter- or intramolecular side-transesterification reaction [9,13]. However, the polymerization of cyclic monomers by the ROP is free of these limitations and thus is preferred for synthesis of biomedical polyesters [14]. Many metal catalytic systems, such as Al, Ca, Fe, La, Mg, Nd, Sm, Sn, Ti, Zn, Zr, Y and Yb derivatives, are imparting control to the lactones or lactides polymerization process. The ROP of cyclic monomers in the presence of these catalytic systems is a controlled process that provides polyesters with narrow *PD* [14,15].

Recently, the polymerization activity of various Zn/ligand catalytic systems has been studied [13]. Calixarenes, alkoxides, diamino phenolates, beta-diketiminates, phosphinophenolates, Schiff-base macrocycles, aryloxides, anilido imines, dialkoxo/diamines, phenoxy imines, heteroscorpionates, phosphinimino methanides, tripodal triamines, and pyridine imines have been used as ligands in the synthesis of coordination zinc catalysts [13]. However, to the best of our knowledge, there is no report on the application of Zn/gallic acid or Zn/propyl gallate as catalytic systems of the ROP of CL.

For safety reasons, any residual metal content must be removed from the final materials before use in pharmaceutical or medical applications [14]. Another approach for producing biomedical polymers free of toxic metallic residues uses bioresorbable salts (e.g., Na^+^, K^+^, Mg^2+^, Ca^2+^, Zn^2+^, and Fe^2+^) for the ROP of cyclic esters. For example, zinc lactate gives good toxicological results [14,16,17,18,19], whereas zinc acetate turns out to be a friendly analogue of tin(II) 2-ethylhexanoate [14,20]. 

In the present paper we describe a new and effective synthesis of PCL. It involves the ROP of CL in the presence of new catalytic systems: diethylzinc/gallic acid and diethylzinc/propyl gallate. We believe that the thus-obtained PCLs could be practically applied in drug delivery systems.

## 2. Results and Discussion

**Figure 1:**
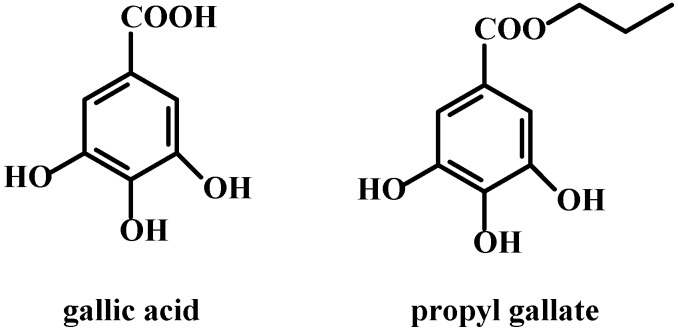
Structure of gallic acid and propyl gallate.

**Scheme 1:**
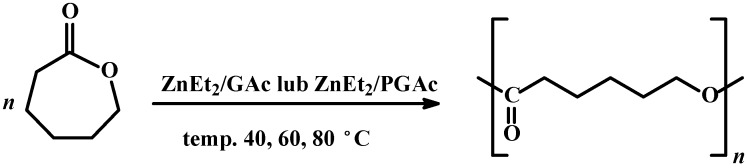
Ring opening polymerization of CL in the presence of ZnEt_2_/GAc or ZnEt_2_/PGAc catalytic systems.

The effect of various parameters on the yield of ROP of CL and the average molecular weight of obtained PCL using new Zn-based catalysts has been studied. The homopolymerization of CL was carried out in the presence of the ZnEt_2_/GAc or ZnEt_2_/PGAc catalytic systems at 40–80 °C within 6–48 h (Figure 1, Scheme 1). The catalytic systems were obtained in the reaction of ZnEt_2_ with GAc (or PGAc) at a molar ratio of 3:1. Toluene was used as the solvent. 

The molar ratio of the catalyst ([Zn]_0_) to a given monomer was 1:50 or 1:100. The reaction yields and the average molecular weight of the obtained PCLs are summarized in Table 1.

**Figure 2:**
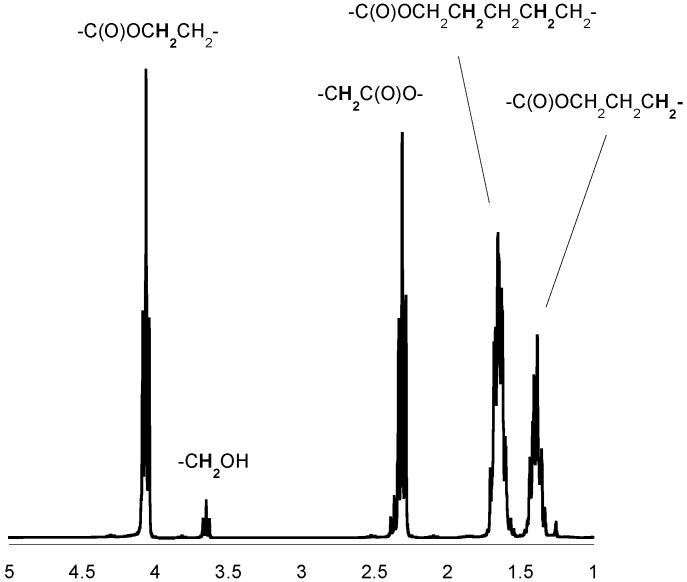
^1^H-NMR spectrum of the PCL (in CDCl_3_).

**Figure 3:**
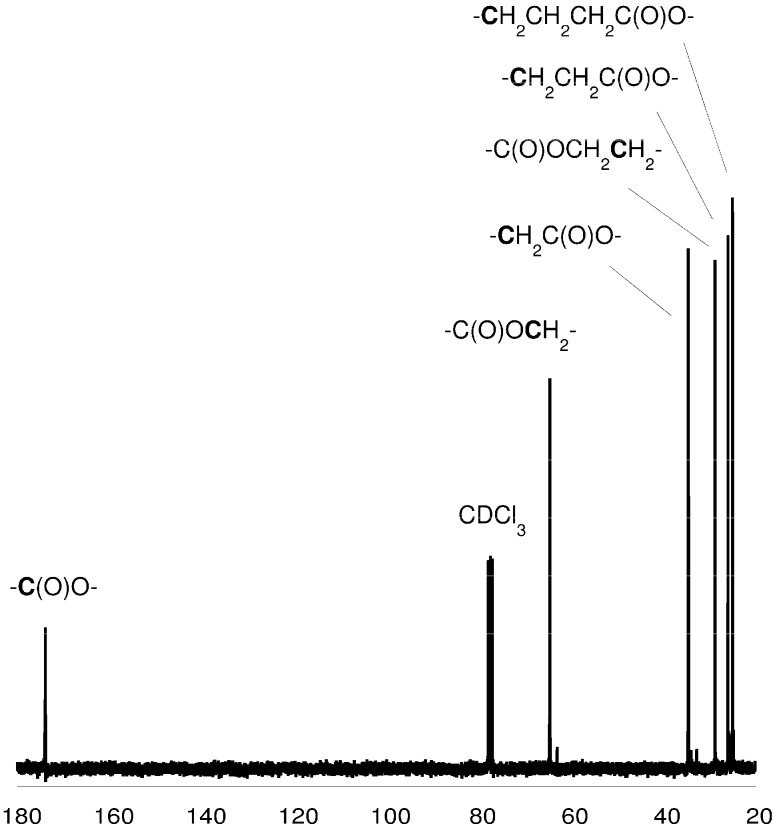
^13^C-NMR spectrum of the PCL (in CDCl_3_).

The structure of the obtained PCLs was confirmed by ^1^H- or ^13^C-NMR and FTIR techniques (see Experimental Section). Figure 2 and Figure 3 show typical spectra of the synthesized polymeric products. 

**Table 1:** Homopolymerization of CL in the presence of ZnEt_2_/GAc and ZnEt_2_/PGAc catalytic systems.

Entry	Catalytic System	Molar Ratio [Zn]_0_: [CL]_0_	Temp. (°C)	Yield (%)	Conv. ^a^ (%)	*M_n_* ^b^ [Da]	*PD* ^b^	*MC* ^c^ (%)	*M_v_* ^d^ [Da]	*M_n_* ^e^ [Da]
**PCL-1**	ZnEt_2_/GAc	1/50	40	45	48	1900	1.40	0	2200	1700
**PCL-2**	ZnEt_2_/GAc	1/100	40	49	53	2900	1.51	0	3100	2500
**PCL-3**	ZnEt_2_/GAc	1/50	60	60	64	3100	1.59	18	3900	2900
**PCL-4**	ZnEt_2_/GAc	1/100	60	54	59	5900	1.73	14	6400	5300
**PCL-5**	ZnEt_2_/GAc	1/50	80	63	69	3600	1.61	54	4000	3100
**PCL-6**	ZnEt_2_/GAc	1/100	80	59	65	6200	1.78	43	5000	5700
**PCL-7**	ZnEt_2_/PGAc	1/50	40	47	52	2300	1.39	0	3100	2200
**PCL-8**	ZnEt_2_/PGAc	1/100	40	72	79	7900	1.78	0	7000	7400
**PCL-9**	ZnEt_2_/PGAc	1/50	60	71	78	4500	1.67	0	5400	4200
**PCL-10**	ZnEt_2_/PGAc	1/100	60	90	100	10,500	1.82	0	12,400	9500
**PCL-11**	ZnEt_2_/PGAc	1/50	80	84	92	4700	1.71	7	6100	4600
**PCL-12**	ZnEt_2_/PGAc	1/100	80	96	100	12,300	1.89	5	10,800	9900
**PCL-13**	ZnEt_2_/GAc *	1/100	80	32	44	3300	1.48	9	3900	3100
**PCL-14**	ZnEt_2_/GAc **	1/100	80	47	56	4800	1.57	14	4700	4100
**PCL-15**	ZnEt_2_/PGAc *	1/100	80	67	74	8100	1.69	0	7200	6500
**PCL-16**	ZnEt_2_/PGAc **	1/100	80	85	93	9300	1.82	3	8700	8200
**C-1**	ZnEt_2_	1/100	40	traces	-	-	-	-	-	-
**C-2**	ZnEt_2_	1/100	60	28	34	2900	1.92	38	3300	2800
**C-3**	ZnEt_2_	1/100	80	39	48	3700	2.35	56	4200	3900
**C-4**	ZnEt_2_ *	1/100	80	traces	-	-	-	-	-	-
**I-1**	GAc	1/100	80	0	0	-	-	-	-	-
**I-2**	PGAc	1/100	80	0	0	-	-	-	-	-

Reaction conditions: argon atmosphere, reaction time—48 h (* 6 h, ** 12 h); ^a^ calculated from ^1^H NMR analysis; ^b^ determined by GPC; *M_n_* corrected by a factor of *ca.* 0.47 [21,22]; ^c^ *MC* (macrocyclic content) determined by MALDI TOF MS; ^d^ determined by viscosity method (K = 1.94·10^−4^ dL/g and α = 0.73) [23]; ^e^ determined by ^1^H-NMR.

**Figure 4:**
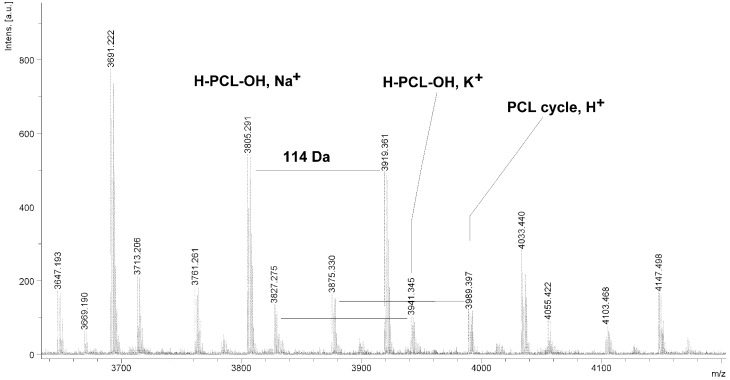
MALDI TOF MS spectrum of PCL obtained in the presence ZnEt_2_/GAc catalytic system (**PCL-3**).

**Figure 5:**
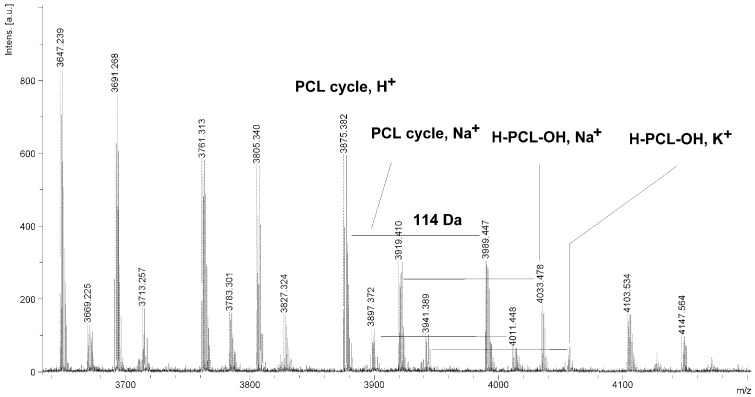
MALDI TOF MS spectrum of PCL obtained in the presence ZnEt_2_/GAc catalytic system (**PCL-5**).

**Figure 6:**
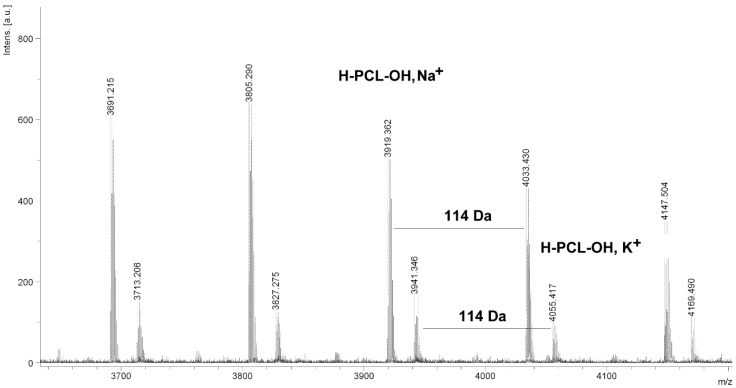
MALDI TOF MS spectrum of PCL obtained in the presence ZnEt_2_/PGAc catalytic system (**PCL-9**).

**Figure 7:**
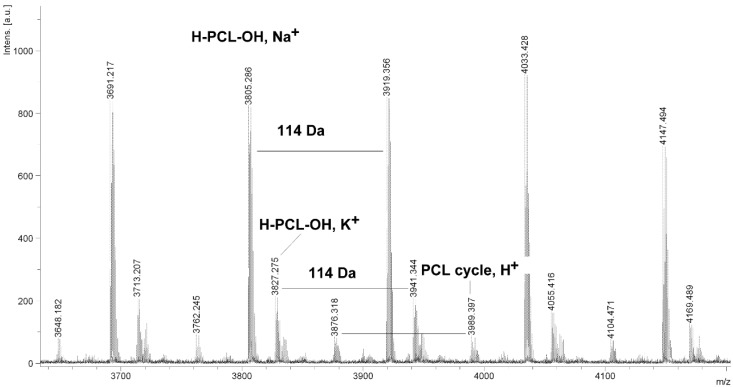
MALDI TOF MS spectrum of PCL obtained in the presence ZnEt_2_/PGAc catalytic system (**PCL-11**).

The MALDI TOF MS spectra of PCL contain several peaks; each component corresponds to a separate spectrum series (Figure 4, Figure 5, Figure 6 and Figure 7). The first series of peaks is characterized by a mass increment of 114 Da, which is equal to the mass of the repeating CL unit in the PCL. It can be assigned to PCL terminated with a hydroxyl group (residual mass: *ca*. 41 Da, Na^+^ adduct; **H-PCL-OH**, **Na^+^**). The peaks of the second population may be also attributed to PCL terminated with a hydroxyl group (residual mass: *ca*. 57 Da, K^+^ adduct; **H-PCL-OH**, **K^+^**). The third and fourth series of peaks are probably from macrocyclic CL homopolymers (residual mass: *ca*. 1 Da, H^+^ adduct; **PCL cycle**, **H^+^** or 23 Da, Na^+^ adduct; **PCL cycle**, **Na^+^**). The content of cyclic products (*CP*) was estimated on the basis of the intensity ratio of the peaks for linear and cyclic PCL. The *CP* was generally increased with increasing temperature and polymerization time. The results show that under the process conditions the polymer chain undergoes intramolecular transesterification (leading to the formation of *CP*), which is a typical phenomenon for the polymerization of cyclic monomers.

It is worth mentioning that the macrocyclic content (*MC*) for PCLs obtained in the presence of a ZnEt_2_/PGAc catalytic system is apparently small when compared to the *MC* for PCLs obtained in the presence of a ZnEt_2_/GAc catalytic system (Table 1). For example, the *MC* value was 18% for **PCL-3**, 54% for **PCL-5**, 0% for **PCL-9** and 7% for **PCL-11**, respectively. When, the ROP of the CL process was carried out in the presence of ZnEt_2_/GAc catalytic system at 40 °C within 48 h, *CP* were not formed. For comparison, the *MC* was 0% for the reaction carried out in the presence of a ZnEt_2_/PGAc catalytic system at 40–60 °C within 48 h or at 80 °C within 6 h. However, on the basis of the intensity of **H-PCL-OH** peaks for products obtained in the presence of a ZnEt_2_/PGAc catalytic system, it can be estimated that the content of *CP* does not exceed 14 mol %.

The yield of the ROP process ranged from 32% to 63% for ZnEt_2_/GAc and from 47% to 96% for ZnEt_2_/PGAc catalytic systems. For ZnEt_2_/GAc and ZnEt_2_/PGAc the corresponding monomer conversion values were in the range of 44%–69% and 52%–100%, respectively. The yield of the CL polymerization increased with increasing the reaction temperature and time (Table 1). For example, the reaction yields were as follow 72% (the reaction temp. 40 °C, catalyst/monomer feed ratio 1:100), 90% (60 °C, 1:100) and 96% (80 °C, 1:100) for **PCL-8**, **PCL-10** and **PCL-12**, respectively. When the process was carried out in the presence of (ZnEt_2_/PGAc) catalytic systems at the molar ratio of catalyst to monomer 1:100 (reaction temp. 80 °C), the reaction yields were: 67% for **PCL-15** (reaction time 6 h), 85% for **PCL-16** (reaction time 12 h) and 96% for **PCL-12** (reaction time 48 h).

The viscosity molecular mass (*M_v_*) values for PCLs were in the range of 2200–6400 Da (for ZnEt_2_/GAc) and 3100–12,400 Da (ZnEt_2_/PGAc). For comparison, *M_n_* determined from GPC for PCLs obtained in the presence of ZnEt_2_/GAc or ZnEt_2_/PGAc was in the range of 1900–6200 Da (*PD* 1.40–1.78) and 2300–12,300 Da (*PD* 1.39–1.89), respectively. The GPC values were adequately corrected using a factor of 0.47 [21,22].

As shown in Table 1, the average molecular mass of PCL is dependent on the Zn-based catalysts/CL molar ratio and the reaction time. The influence of the catalyst/monomer feed ratio on the average molecular weight of PCL was studied at two levels (1:50 and 1:100). It was found that the average molar mass of polymers increased with a feed ratio of Zn/CL decrease. For example, the *M_n_* determined from GPC was 4500 Da for **PCL-9** (molar ratio 1:50) and 10,500 Da for **PCL-10** (molar ratio 1:100). 

As shown in Table 1, the *M_n_* values calculated from NMR were consistent with the *M_n_* determined by GPC. This indicated that the *M_n_* of the obtained PCLs can be relatively well controlled by the molar ratio of monomer to catalysts systems. 

The CL monomer was completely consumed within 48 h at 60 or 80 °C, when the ROP process was carried out in the presence of ZnEt_2_/PGAc catalytic system. In comparison, the maximum conversion for ROP catalysed by ZnEt_2_/GAc was 69% within 48 h at 80 °C. This indicates that ZnEt_2_/PGAc is a more effective catalytic system in promoting the polymerization of CL than ZnEt_2_/GAc. This is probably a result of the fact that in the first case only alcoholate active species are formed, whereas in the second case carboxylate species are also formed. 

As initiated by other metal alkoxides, the ROP of CL catalysed by ZnEt_2_/GAc or ZnEt_2_/PGAc probably follows a coordination-insertion mechanism. The reaction proceeds through cleavage of the acyl-oxygen bond of CL and insertion of the lactone between zinc and oxygen atoms. However, it is difficult to obtain molecular zinc complexes (in the reaction of ZnEt_2_ with GAc or PGAc) because of the strong association tendency of products in the reaction medium.

For comparison, ROP of CL has been carried out in the presence of only ZnEt_2_ or GAc/PGAc. As shown in Table 1, GAc and PGAc have not initiated ROP of CL under process conditions (Figure S1, Supplementary Materials). The traces of polymeric product have only been observed, when the ROP has been carried out in the presence of ZnEt_2_ at 40 °C (48 h) or 80 °C (6 h) (Figure S2, Supplementary Materials). When, the ROP of CL was performed in the presence of ZnEt_2_ at 60 °C or 80 °C within 48 h, the yield of the product was 28% and 39%, respectively (Figure S3, Supplementary Materials). Moreover, the *CP* were intensively formed (38%–56%) (Figure S4, Supplementary Materials). We assume, that in this case, the initiation process of ROP was most certainly affected by water traces from the reaction mixture. The zinc alkoxide-active centres were generated from the reaction of ZnEt_2_ with water, and then this product initiated ROP of CL [6]. Unfortunately, it was impossible to completely remove water from the substances and solvent to avoid this process.

## 3. Experimental Section 

### 3.1. Materials

All substances were purified, stored and used in dry argon atmosphere. ε-Caprolactone (2-oxepanone, 99%, CL, Sigma-Aldrich, Co., Poznan, Poland) was dried and distilled over CaH_2_ at reduced pressure on dried 5 A° molecular sieves before use. Toluene (anhydrous, 99.8%, POCh, Gliwice, Poland) and 1,4-dioxane (anhydrous, 99.8%, Sigma-Aldrich, Co.) were fractionally distilled from sodium/-potassium and benzophenone after colour change to navy blue, and then stored over dried 4 A° molecular sieves. Dichloromethane (anhydrous, >99.8%, CH_2_Cl_2_, POCh) was fractionally distilled from CaH_2_ onto dried 4 Å molecular sieves. Diethylzinc (solution 15 wt. % in toluene, ZnEt_2_, Sigma-Aldrich, Co.), gallic acid (3,4,5-trihydroxybenzoic acid, 97.5%–102.5%, GAc, Sigma-Aldrich, Co.), and propyl gallate (3,4,5-trihydroxybenzoic acid propyl ester, ≥98%, PGAc, Sigma-Aldrich, Co.) were used as received. 

### 3.2. Synthesis of Catalytic Systems

The catalytic systems were prepared in an argon atmosphere at room temperature immediately before each reaction. The reactions were carried out in 100-mL three-necked round-bottomed flasks. Each glass vessel was equipped with a magnetic stirrer. The flask was contained a mixture of ZnEt_2_ (0.0177 mol) and GAc (or PGAc) (0.0059 mol) in a molar ratio of 3 to 1 and toluene (or 1,4-dioxane) as a solvent (35 mL). The content of the flask was vigorously stirred. The reactions were carried out for about 2 h. The volume of ethane evolved was measured in a gas burette.

### 3.3. Polymerization Procedure

The ROP of CL was carried out in a 10 mL glass tube. The required amount of monomer and ZnEt_2_/GAc or ZnEt_2_/PGAc catalytic systems were placed in a 10 mL polymerization glass ampoules under argon atmosphere. The reaction vessel was then kept standing in an oil bath kept at an appropriate temperature using a thermostat (40, 60 or 80 °C) for the required time. Then, the polymerization products were cooled down and dissolved in CH_2_Cl_2_. The obtained solutions were washed with dilute hydrochloric acid (5% aqueous solution) and distilled water (three times). Next, precipitated polymers were dried at room temperature in a vacuum for 2–3 days.

### 3.4. Measurements

Relative molecular mass and molecular mass distribution were determined by GPC, MALDI-TOF or viscosity techniques. GPC instrument (GPC Max + TDA 305, Viscotek, Malvern, UK) was equipped with Jordi DVB Mixed Bed columns (one guard and two analytical) at 30 °C in CH_2_Cl_2_ (HPLC grade, Sigma-Aldrich), at a flow rate of 1 mL/min with RI detection and calibration based on narrow PS standards (ReadyCal Set, Fluka, Poznan, Poland). Results were processed with OmniSEC software (ver. 4.7). The MALDI TOF mass spectra were performed in linear mode on an ultrafleXtreme™ (Bruker Daltonics, Poznan, Poland) mass spectrometer using a nitrogen gas laser and DCTB as a matrix. The PCL samples were dissolved in THF (5 mg/mL) and mixed with a solution of DCTB. PCL viscosity was measured in *N*,*N*-dimethylformamide (DMF) (at 30 °C) on a Stabinger Viscometer SVM 3000 (Anton Paar’s, Graz, Austria). The concentrations of the PCL solutions in DMF were 0.2%, 0.4%, 0.6%, 0.8% and 1%. The viscosity average molecular weight was calculated with the Mark-Houwink equation using the following constants: K = 1.94·10^−^^4^ dL/g and α = 0.73 [23]. The polymerization products were characterized by means of ^1^H- or ^13^C-NMR (Varian UNITY 300 MHz, Palo Alto, CA, USA, recorded in CDCl_3_) and FTIR spectroscopy (PerkinElmer, Waltham, MA, USA). FTIR spectra were measured using KBr pellets. 

### 3.5. *Spectroscopic Data:* Poly(ε-caprolactone) 

^1^H-NMR (CDCl_3_, δ, ppm): 4.04 [2H, t, -CH_2_CH_2_CH_2_CH_2_C**H_2_**OC(O)-], 2.28 [2H, t, -CH_2_CH_2_CH_2_CH_2_C**H_2_**COO-], 1.62 [4H, m, -CH_2_C**H_2_**CH_2_C**H_2_**CH_2_COO-], 1.37 [2H, m, -CH_2_CH_2_C**H_2_**CH_2_CH_2_COO-]. ^13^C-NMR (CDCl_3_, δ, ppm): 173.2 [-**C**(O)O-], 63.8 [-CH_2_CH_2_CH_2_CH_2_**C**H_2_OC(O)-], 33.7 [-CH_2_CH_2_CH_2_CH_2_**C**H_2_COO-], 28.0 [-CH_2_CH_2_CH_2_**C**H_2_CH_2_OC(O)-], 25.3 [-CH_2_CH_2_CH_2_**C**H_2_CH_2_COO-], 24.3 [-CH_2_CH_2_**C**H_2_CH_2_CH_2_COO-]; FTIR (KBr, cm^−1^): 2943 (ν_as_CH_2_), 2866 (ν_as_CH_3_), 1723 (νC=O), 1243 (νC-O).

## 4. Conclusions

In this study, we have reported a new and effective zinc-based catalytic system for the ring-opening polymerization (ROP) of ε-caprolactone (CL). The catalytic systems were prepared from diethylzinc and gallic acid (ZnEt_2_/GAc) or propyl gallate (ZnEt_2_/PGAc) for the first time. Interestingly, the ZnEt_2_/GAc or ZnEt_2_/PGAc catalytic systems were quite effective in the ROP of CL. Polymerization in bulk at 40–80 °C produced polyesters with a high yield (even *ca.* 100% in some cases). It was also found that the efficiency of the ZnEt_2_/GAc catalytic system was lower when compared to ZnEt_2_/PGAc catalytic system. Most importantly, when the ROP of CL was carried out in the presence of ZnEt_2_/PGAc catalytic system at 40–60 °C within 48 h or at 80 °C within 6 h, the macrocyclic products were not formed. The obtained results are promising for application of the synthesized polymers as drug carriers. 

## Supplementary Materials

Supplementary materials can be accessed at: http://www.mdpi.com/1420-3049/20/02/2816/s1.
