# Supplementary material for: Novel Zinc-Catalytic Systems for Ring-Opening Polymerization of ε-Caprolactone
Source: Molecules. 2015 Feb 9;20(2):2816–27. doi: 10.3390/molecules20022816 (PMC6272648; doi:10.3390/molecules20022816)
Supplement: Supplementary file 1 [file molecules-20-02816-s001.pdf]

Supplementary Materials

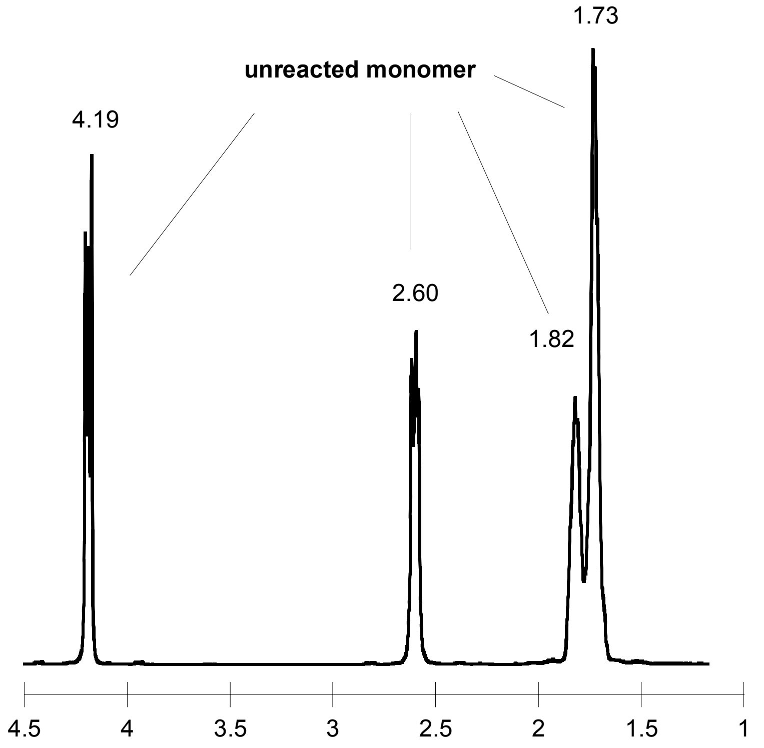

Figure S1. ^{1}H-NMR spectrum of reaction product carried out in the presence of PGAc (in $CDCl_3$) (**I-2**).

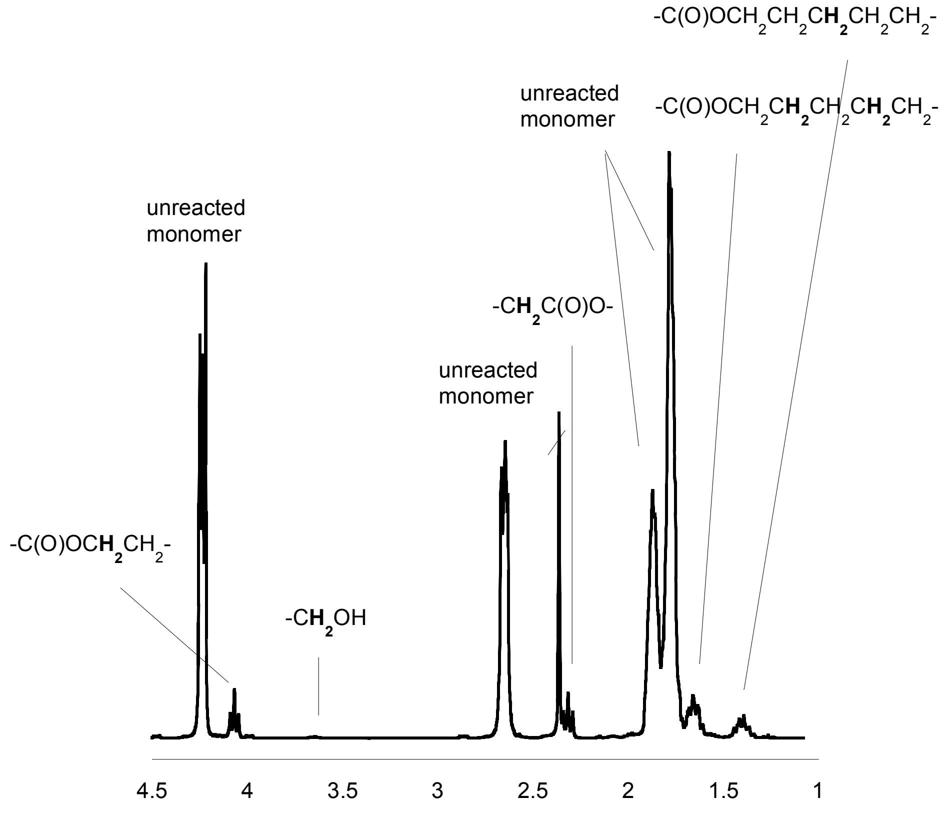

Figure S2. ^{1}H-NMR spectrum of reaction product carried out in the presence of $ZnEt_2$ at 40 °C (in $CDCl_3$) (**C-1**).

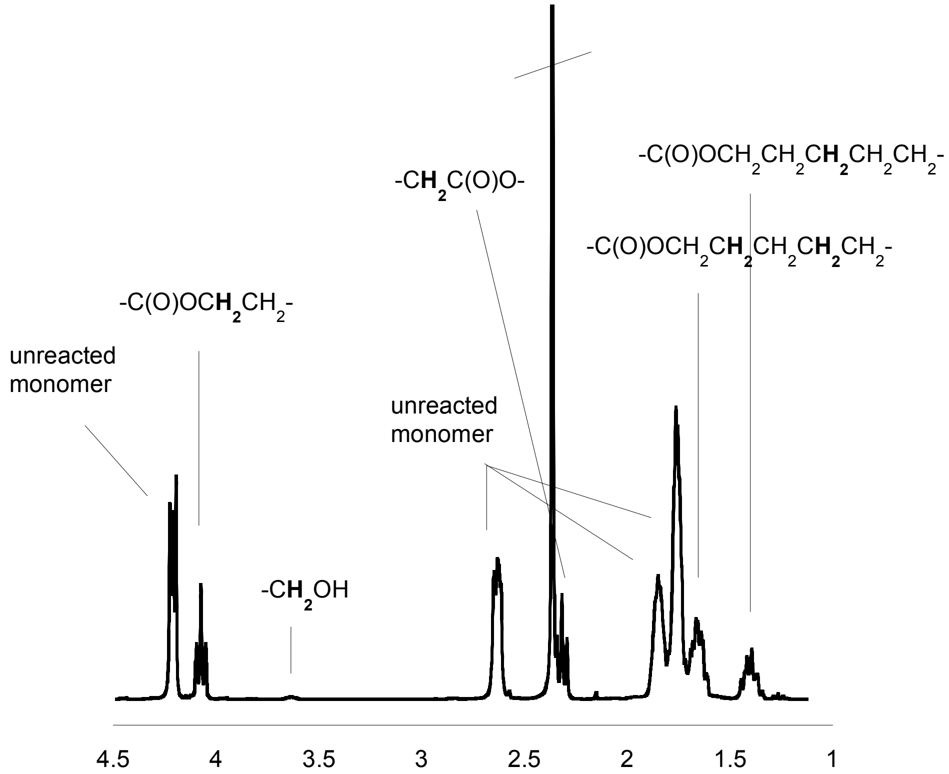

Figure S3. ^{1}H-NMR spectrum of reaction product carried out in the presence of $ZnEt_2$ at 60 °C (in $CDCl_3$) (**C-2**).

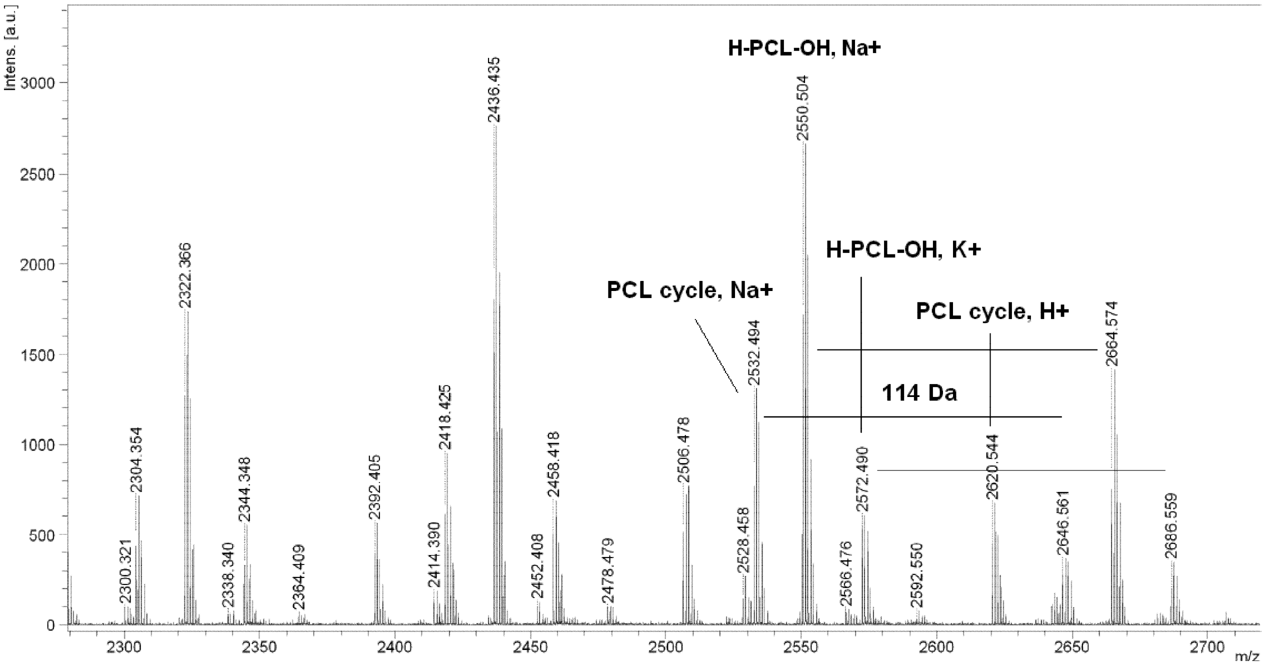

Figure S4. MALDI TOF MS spectrum of PCL obtained in the presence of $ZnEt_2$ at 60 °C (**C-2**).
